# Discovery and pharmacological characterization of cetrelimab (JNJ-63723283), an anti–programmed cell death protein-1 (PD-1) antibody, in human cancer models

**DOI:** 10.1007/s00280-022-04415-5

**Published:** 2022-03-17

**Authors:** Nikki DeAngelis, Catherine Ferrante, Gordon Powers, Jocelyn Sendecki, Bethany Mattson, Darlene Pizutti, Kathryn Packman, Weirong Wang, Kevin Trouba, Rupesh Nanjunda, John Wheeler, Ray Brittingham, Sheng-Jiun Wu, Jinquan Luo, Matthew V. Lorenzi, Raluca I. Verona

**Affiliations:** 1 Oncology Therapeutic Area, Janssen Research & Development, Spring House, PA USA; 2 Biologics Research, Janssen Research & Development, Spring House, PA USA; 3 Translational Medicine and Early Development Statistics, Janssen Research & Development, Spring House, PA USA; 4 Biologics Development Sciences, Janssen Research & Development, Spring House, PA USA; 5 Nonclinical Safety, Janssen Research & Development, Spring House, Pennsylvania USA

**Keywords:** Immune checkpoint inhibitor, Programmed cell death protein-1, T cell function, Antitumor activity, Toxicokinetic assessment

## Abstract

**Purpose:**

Preclinical characterization of cetrelimab (JNJ-63723283), a fully humanized immunoglobulin G4 kappa monoclonal antibody targeting programmed cell death protein-1 (PD-1), in human cancer models.

**Methods:**

Cetrelimab was generated by phage panning against human and cynomolgus monkey (cyno) PD-1 extracellular domains (ECDs) and affinity maturation. Binding to primate and rodent PD-1 ECDs, transfected and endogenous cell-surface PD-1, and inhibition of ligand binding were measured. In vitro activity was evaluated using cytomegalovirus recall, mixed lymphocyte reaction, staphylococcal enterotoxin B stimulation, and Jurkat-PD-1 nuclear factor of activated T cell reporter assays. In vivo activity was assessed using human PD-1 knock-in mice implanted with MC38 tumors and a lung patient-derived xenograft (PDX) model (LG1306) using CD34 cord-blood-humanized NSG mice. Pharmacodynamics, toxicokinetics, and safety were assessed in cynos following single and/or repeat intravenous dosing.

**Results:**

Cetrelimab showed high affinity binding to human (1.72 nM) and cyno (0.90 nM) PD-1 and blocked binding of programmed death-ligand 1 (PD-L1; inhibitory concentration [IC] 111.7 ng/mL) and PD-L2 (IC 138.6 ng/mL). Cetrelimab dose-dependently increased T cell-mediated cytokine production and stimulated cytokine expression. Cetrelimab 10 mg/kg reduced mean MC38 tumor volume in PD-1 knock-in mice at Day 21 (*P* < 0.0001) versus control. In a PDX lung model, 10 mg/kg cetrelimab (every 5 days for six cycles) increased frequency of peripheral T cells and reduced (*P* < 0.05) mean tumor volume versus control. Activity was consistent with that of established PD-1 inhibitors. Cetrelimab dosing was well tolerated in cynos and mean drug exposure increase was dose-dependent.

**Conclusion:**

Cetrelimab potently inhibits PD-1 in vitro and in vivo, supporting its clinical evaluation.

## Introduction

Programmed cell death protein-1 (PD-1) is a negative immune checkpoint receptor expressed on CD4^+^ and CD8^+^ T cells. In healthy individuals, binding of PD-1 and its ligands programmed death-ligand 1 (PD-L1) and 2 (PD-L2) contributes to immune tolerance by regulating T cell activation [1–3]. In cancer, transformed cells exploit this pathway to evade immune surveillance; in the tumor microenvironment, persistent antigen exposure and inflammation drive T cell exhaustion, a state in which T cells progressively lose effector function and proliferative capacity [3, 4]. T cell exhaustion is characterized by PD-1-mediated suppression of anti-inflammatory cytokine release and T cell receptor (TCR) signaling [5] and was found to be reversed by blockade of PD-1 binding to its ligand [4, 6–8] in cell cultures and animal models [5, 9].

In the clinic, disruption of the PD-1/PD-L axis using monoclonal antibodies (mAbs) directed at either the PD-1 receptor or ligands PD-L1 and PD-L2 has proven to be an effective and well-tolerated therapy for a range of tumor types such as metastatic melanoma, non-small-cell lung cancer, head and neck carcinoma, classic Hodgkin lymphoma, urothelial and renal carcinoma, cutaneous squamous carcinoma, and solid tumors confirmed to be microsatellite instability high (MSI-H) or mismatch repair deficient (dMMR) [10, 11]. Despite these successes, durable responses are achieved in only a subset of indicated patients [12–17]. Treatment has also been associated with nonresponses, immune tolerance, and elevated toxicity [18]. These findings have prompted continued development of additional anti-PD-1 agents for use alone or in combination with other treatments to provide efficacy to a broader patient population.

Here we report the discovery and pharmacologic characterization of cetrelimab (JNJ-63723283), an anti-PD-1 antibody currently in clinical development. In preclinical human models, we assessed cetrelimab activation of T cells, reversal of PD-1 suppression of TCR signaling, and in vivo antitumor efficacy. We compared the activity of cetrelimab with that of the established PD-1 inhibitors pembrolizumab and nivolumab (analogs and commercially available material). The results presented here support the ongoing phase 1/2 study (NCT02908906, EudraCT2016-002017–22) of cetrelimab in patients with solid tumors; two phase 2 studies (SunRISe-1 [NCT04640623, EudraCT2020-002646–16] and SunRISe-4 [NCT04919512, EudraCT2020-005565–13]) of cetrelimab in combination with TAR-200 in patients with non-muscle invasive bladder cancer (NMIBC) and muscle-invasive bladder carcinoma (MIBC), respectively; and phase 3 study (SunRISe-2, NCT04658862, EudraCT2020-002620–36) of cetrelimab in combination with TAR-200 versus concurrent chemoradiotherapy in patients with MIBC.

## Materials and methods

### Antibody generation

Cetrelimab, a fully human immunoglobulin (Ig)G4 kappa mAb containing the hinge stabilizing *S228P* mutation [19], was generated by phage panning of de novo pIX Fab libraries [20] against various forms of recombinant PD-1 extracellular domain (ECD) proteins. HumanPD1His (HumanPD1HisTagged; Catalog #10377-H08H-B, Sino Biological, Wayne, PA), HumanPD1Fc (HumanPD1FcTagged; Catalog#1086-PD, R&D Systems, Inc., Minneapolis, MN), MurinePD1Fc (MurinePD1FcTagged; Catalog#1021-PD, R&D Systems), and/or CynoPD1Fc (Janssen, Spring House, PA) were all used in various formats for selections. The recombinant proteins were biotinylated and captured on streptavidin magnetic beads [Dynal, DynaBeads M280; Catalog #10006D Thermo Fisher Scientific (Invitrogen), Waltham, MA], then exposed to the de novo pIX Fab libraries. Nonspecific phages were washed away in phosphate-buffered saline (PBS) with 0.05% (weight/volume) Tween 20 and bound phages were recovered by infection of MC1061F′ *E. coli* cells. Following the final round of panning, monoclonal Fab was screened for binding to either HumanPD1His, HumanPD1Fc, MurinePD1Fc, and/or CynoPD1Fc in ELISA. Clones that demonstrated binding to the proteins were sequenced in the heavy and light chain variable regions. Clones having desired affinity, epitope, and activity profiles were affinity matured to increase their binding affinities to human and cynomolgus monkey (cyno) PD-1. Affinity maturation libraries altering potential PD-1 contact amino acid residues in the antibody complementarity-determining regions were constructed and phage was generated. The phage libraries were then used for phage panning against human PD-1 and cyno PD-1 biotinylated recombinant proteins. Following phage panning, soluble Fabs were screened for binding affinity to both human and cyno PD-1.

### PD-1 binding profile

#### Affinities for PD-1 ECD

Cetrelimab binding to human (Sino Biological), cyno (Janssen), rat (Sino Biological), and mouse (Sino Biological) PD-1 ECDs was assessed using ProteOn surface plasmon resonance analysis (Bio-Rad, Hercules, CA). Cross-reactivity to other human PD-1 family members (MOG [Sino Biological]), VISTA [Sino Biological], B7-H4, B7-H7, B7-H6, B7-H1, B7-H3, and PD-L2 [all R&D Systems]) was similarly assessed. Goat anti-human and anti-mouse Fc capture antibodies were amine coupled to GLC type ProteOn sensor chips. Anti-PD-1 mAbs and Fc-fused PD-1 family member proteins were subsequently captured by the antibodies on the chips. PD-1 family member proteins (1600–6.3 nM at fourfold dilutions in phosphate-buffered saline tris-ethylenediaminetetraacetic acid) were injected horizontally over captured anti-PD-1 mAb for the monitoring of association and dissociation kinetics for 4 and 30 min, respectively. In the case of ligands where monomeric versions were not available, the Fab of cetrelimab was injected over Fc-captured PD-1 family member proteins.

#### Affinity for cell-surface PD-1

Human embryonic kidney (HEK293) cells (1 × 10^5^ cells) stably overexpressing cyno PD-1 (DD16339 11 HEK293; generated at Janssen) were incubated with increasing concentrations of cetrelimab. Binding was detected by flow cytometry using R-phycoerythrin conjugated goat anti-human IgG, F(ab′)2 fragment (Jackson ImmunoResearch, West Grove, PA; Catalog #109–116-097) and measured with the MACSQuant Analyzer 10 (Miltenyi Biotec, San Diego, CA).

#### Ligand binding inhibition

The ability of cetrelimab or IgG4 isotype control to block binding of PD-1 to its ligands, PD-L1 and PD-L2, was assessed at concentrations of 1, 10, 100, and 1000 ng/mL. Test antibodies were incubated with biotinylated human PD-1, and subsequent binding to plate-bound human PD-L1 or human PD-L2 ligands was measured using Meso Scale Discovery 6000 platform (Meso Scale Diagnostic, LLC, Rockville, MD).

#### Binding to activated human T cells

Activation of pan-T cells (HemaCare Donor #PB03C3; Lot 033, HemaCare Corporation, Northridge, CA) was done using Perform CD3/CD28 beads. On Day 6, activator beads were removed, and cells were treated with Fc block (TruStain FcX BioLegend; San Diego, CA, Catalog #422301) and incubated in the presence of 1:3-fold titrations of cetrelimab or isotype control. Binding was quantified using a MACSQuant Analyzer after incubation with phycoerythrin-labeled (PE) anti-human PD-1 (BioLegend, Catalog #329906), or PE murine IgG1/κ isotype control (BioLegend, Catalog #400112).

### In vitro assays

#### Mixed lymphocyte reactions

Cetrelimab, as well as pembrolizumab and nivolumab analogs (produced in house based on publicly available sequences), were tested for their ability to enhance T cell function in mixed lymphocyte reaction (MLR) assays. Human CD4^+^ T cells were isolated from peripheral blood mononuclear cells (PBMCs) from healthy donors (Biological Specialty Corporation, Colmar, PA) using a CD4^+^ isolation kit (Miltenyi Biotec, San Diego, CA, Catalog #130–096-533). Purified human T cells (1.5 × 10^5^ cells) were activated by stimulation with allogeneic, major histocompatibility complex (MHC)-mismatched, dendritic cells (5 × 10^3^ cells; HemaCare) for 5 days in the presence of cetrelimab, pembrolizumab analog, nivolumab analog (final concentration: 30, 10, 3.3, 1.1, 0.36, 0.12, 0.04, 0.01, 0.004, 0.001 nM), or isotype control (Janssen).

#### Specific antigen recall

In the cytomegalovirus (CMV) recall assays, CMV-reactive T cells (1.5 × 10^5^ cells; Astarte Biologics, Bothell, WA) extracted from peripheral blood of CMV-responsive donors were stimulated with CMV antigen (Astarte Biologics, Catalog #1004) for 6 days in the presence of cetrelimab at concentrations of 0.001, 0.01, 0.1, 1.0, 10.0, and 100 nM. Isotype-control antibody (Janssen) was tested at 30 nM. Cell culture supernatants were analyzed for cytokines using Meso Scale Discovery platform. Preliminary data were analyzed in Spotfire version 5.5.0 using R Service Bus (Open Analytics Antwerp, Belgium).

#### Staphylococcal enterotoxin B stimulation

PBMCs (2 × 10^5^ cells; HemaCare Corporation, Northridge, CA, Catalog #PB009C-1) from two healthy human donors were stimulated with 100 ng/mL staphylococcal enterotoxin B (SEB) (Toxin Technology, Sarasota, FL, Catalog #BT202red) and incubated with cetrelimab or isotype control at a concentration of 10 µg/mL. Plates were incubated at 37 °C, 5% CO_2_ for 3 to 6 days, depending upon the specific assay procedure. On the last day of incubation, 100 µL of supernatant was removed and frozen at −80 °C until analyzed. Data were analyzed in Spotfire version 6.5.3 (TIBCO, Palo Alto, CA) using R Service Bus.

#### Reversal of TCR suppression

A Jurkat reporter assay was used to assess the ability of cetrelimab to reverse PD-1-mediated suppression of TCR signaling. Jurkat T cells (7.5 × 10^4^ cells) engineered to stably express human PD-1 and a nuclear factor of activated T cells (NFAT) response element luciferase reporter (Promega, Madison, WI, US Catalog #J1621 or J1625) were incubated with a PD-L1^+^ Chinese hamster ovary (CHO)-K1 cell line (4 × 10^4^ cells; Promega, Madison, WI, US Catalog #J1621 or J1625). In this setting, PD-1/PD-L1 engagement suppresses the transcriptional activation of the NFAT pathway, resulting in reduced reporter activity. Dose response of cetrelimab, pembrolizumab analog, and nivolumab analog (30, 10, 3.3, 1.1, 0.36, 0.12, 0.04, 0.01, 0.004, or 0.001 μg/mL) on luciferase reporter activity was measured using Bio-Glo Luciferase Assay System (Promega, Madison, WI, US Catalog #G7941) at 24 h after incubation of Jurkat-PD-1 NFAT reporter cells with CHO-K1 expressing PD-L1.

### In vivo activity

In vivo pharmacology experiments were carried out in accordance with The Guide for the Care and Use of Laboratory Animals and were approved by the Institutional Animal Care and Use Committee of Janssen R&D (Spring House, PA) or The Jackson Laboratory (Sacramento, CA). Pivotal nonclinical safety studies have been conducted in conformance with Good Laboratory Practice (GLP) 21 Code of Federal Regulations, Part 58, and the principles of the Organisation for Economic Co-operation and Development (OECD) GLP in countries that are part of the OECD Mutual Acceptance of Data process, and include the appropriate documentation.

#### Human PD-1 knock-in mice

PD-1 knock-in (hPD-1KI) mice, in which the mouse PD-1 ECD has been replaced with the human PD-1 ECD, were licensed from Oxford University Innovation (Oxford, UK) and bred at SAGE Laboratories (Boyertown, PA). To assess development and potential immune abnormalities, phenotypic analysis of knock-in mouse splenocytes was performed. In these hPD-1KI mice, human PD-1 ECD is expressed on CD4^+^ and CD8^+^ T cells upon in vitro activation, similar to what is observed for the endogenous mouse PD-1 protein in wild-type mice.

MC38 syngeneic model mouse colon carcinoma cells (MC38) obtained from (Kerafast Inc, Boston, MA, Catalog #ENH204-FP) were implanted into hPD-1KI mice on Day 0. Starting at Day 7 after implantation (tumors were ~ 98 mm^3^), mice received either cetrelimab, pembrolizumab analog, or control twice weekly by intraperitoneal (IP) injection at a dose of 10 mg/kg. Tumor volume was measured twice weekly throughout the study.

#### Patient-derived xenograft lung model

The patient-derived xenograft (PDX) lung model was run at In Vivo Services at The Jackson Laboratory. Nonobese diabetic, severe combined immunodeficiency (*scid*) gamma (NSG) mice humanized with human CD34^+^ hematopoietic stem cells derived from cord blood from two independent donors were implanted with tumor fragments from the PDX lung model LG1306. When tumor volumes reached 60 to 100 mm^3^, mice were randomized and treated with cetrelimab [10 mg/kg, every 5 days for 6 cycles (*n* = 10–11)], commercially available pembrolizumab [10 mg/kg, every 5 days for 6 cycles (*n* = 10–11)], or human IgG4 isotype control (Janssen). Tumor volume was measured twice weekly throughout the study. Blood samples were taken and mice were euthanized on Day 28, 3 days after the last dose of study treatment. Percentages of cells expressing huCD45 and CD3, CD4, and CD8 were determined using flow cytometry. Flow cytometry was also used to assess specific therapeutic anti-PD-1 antibody target engagement by cetrelimab using a competitive anti-PD-1 antibody.

### Pharmacokinetics, toxicity, and immunogenicity of cetrelimab in cynos

In a 57-day non-GLP pharmacokinetics/pharmacodynamics (PK/PD) study of cetrelimab, groups of two male and two female cynos were administered a single intravenous (IV) dose of cetrelimab at doses of 0.1, 1, and 10 mg/kg. Serum samples for toxicokinetic assessment were obtained on Day 1 prior to dosing and 1 and 8 h post dose, and on Days 2, 3, 4, 8, 12, 15, 22, 29, 36, 43, 50, and 57.

In a 5-week GLP toxicity study, cynos (five per sex per group) were injected IV with 0 (control), 10, 30, or 100 mg/kg of cetrelimab once weekly. Assessments continued in two animals per sex per group during a 4-week recovery period. Data from serum samples obtained post dose on Days 1 and 29 were used to calculate the mean toxicokinetic parameters, using noncompartmental analysis. Twenty-four monkeys (three per sex per group) were euthanatized 1 day following the last dose for primary necropsy. The remaining 12 monkeys (two per sex per group) were killed 4 weeks after the last dose for recovery necropsy. Analyses included clinical signs, qualitative food consumption, body weights, veterinary physical examinations, electrocardiograms, ophthalmology, physiologic parameters (blood pressure, heart rate, and respiration rate), clinical pathology parameters (hematology, coagulation, clinical chemistry, and urinalysis), T cell–dependent antibody response to keyhole limpet hemocyanin and tetanus toxoid, flow cytometry (immunophenotyping), toxicokinetic parameters, antidrug antibody (ADA) evaluation, gross necropsy findings, organ weights, and histopathology.

### Statistical analysis

Robust dose–response curve estimations were used to evaluate antibody binding and the potency of PD-1 antibodies in MLR and CMV recall assays [21–24]. Half maximal effective concentration (EC_50_) values were taken from those estimations.

For SEB stimulation assays, fold-change was calculated as treatment response divided by the average SEB-alone response for each treatment by cytokine, plate, and donor. Linear mixed-effects regression models were constructed, with an outcome of fold-change, a fixed effect of treatment and day, and a random effect of donor. All pairwise comparisons between treatments were made post hoc and adjusted using Tukey’s method [25].

Similarly, comparison of mean tumor volumes in the MC38 tumor model was performed using a linear mixed-effects model. A log-rank Mantel–Cox test was used to determine statistically significant differences in animal survival between treatment groups.

In vivo comparison of tumor growth inhibition in the LG1306 PDX lung model was evaluated using a two-way analysis of variance followed by Dunnett’s multiple comparisons test where significance was determined as *P* ≤ 0.05. Statistical significance for flow cytometry data was calculated using a one-way analysis of variance followed by Dunnett’s multiple comparisons test where significance was determined as *P* ≤ 0.05.

## Results

### Cetrelimab binding to human and cyno PD-1

#### PD-1 ECDs

The affinities (± SEM) of cetrelimab for human (*n* = 13 replicates) and cyno (*n* = 3 replicates) PD-1 ECDs were assessed using ProteOn surface plasmon resonance analysis and determined to be 1.72 ± 0.99 and 0.90 ± 0.08 nM, respectively. Cetrelimab did not show specific binding to rat or mouse PD-1 proteins or to any of the other members of the human PD-1 family assessed in these analyses (MOG, VISTA, B7-H4, B7-H7, B7-H6, B7-H1, B7-H3, and PD-L2).

#### Cell-surface PD-1

**Fig. 1:**
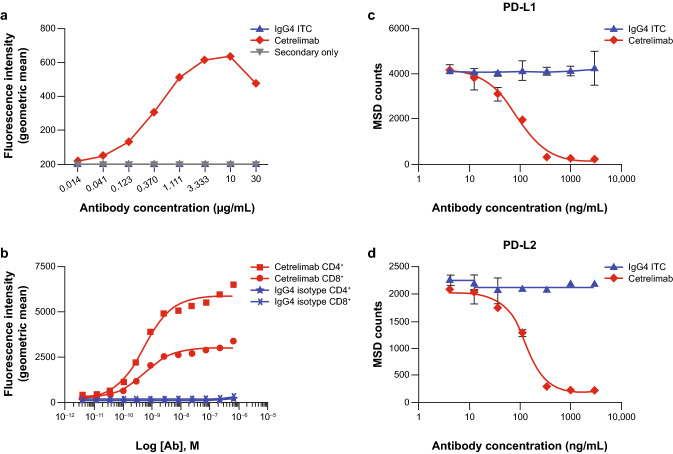
In vitro, cetrelimab binds to PD-1 and blocks PD-1 ligand binding. **a** Cetrelimab binding to HEK-293 cells (1 × 10^5^ cells) overexpressing cyno PD-1. Data are from one experiment. **b** Cetrelimab binding to endogenous PD-1 expressed in activated human CD4^+^ and CD8^+^ T cells. Data are from one experiment. **c** Inhibition of PD-1 binding to human PD-L1. Error bars represent SD from one representative experiment performed in triplicate. **d** Inhibition of PD-1 binding to PD-L2. Error bars represent SD from one representative experiment performed in triplicate. *IgG4 ITC* immunoglobulin G4 isotype control, *MSD* Meso Scale Discovery, *PD-1* programmed cell death protein-1, *PD-L1* programmed death-ligand 1, *PD-L2* programmed death-ligand 2

The capacity of cetrelimab to bind to cell-surface PD-1 was demonstrated in HEK-293 cells overexpressing cyno PD-1 (Fig. 1a). The robust EC_50_ value for cetrelimab was 0.4138 µg/mL.

#### Endogenous PD-1

Cetrelimab bound to endogenous PD-1 on activated cyno CD4^+^ and CD8^+^ T cells from two donors (Fig. 1b). The EC_50_ values for cetrelimab were 0.22 and 0.16 µg/mL on CD4^+^ T cells and 0.22 and 0.17 µg/mL on CD8^+^ T cells for donor 1 and donor 2, respectively.

#### Inhibition of ligand binding

Cetrelimab was tested for its ability to block binding of PD-1 to its ligands, PD-L1 and PD-L2. Cetrelimab completely inhibited binding of PD-1 to both ligands. The cetrelimab IC_50_ (mean ± SD, *n* = 3) was 111.7 ± 22.0 ng/mL for PD-L1 (Fig. 1c) and 138.6 ± 12.4 ng/mL for PD-L2 (Fig. 1d).

### In vitro functional activity

The ability of cetrelimab to promote T cell responses was evaluated in three in vitro assays that measure T cell function upon activation: MLR, SEB, and CMV antigen recall. In these assays, cytokine [tumor necrosis factor-α (TNF-α), interleukin-1β (IL-1β), IL-2, IL-4, IL-5, IL-8, IL-10, IL-12p70, IL-13, interferon-γ (IFN-γ)] production was used as a measure of T cell activity.

#### Mixed lymphocyte reactions

**Fig. 2:**
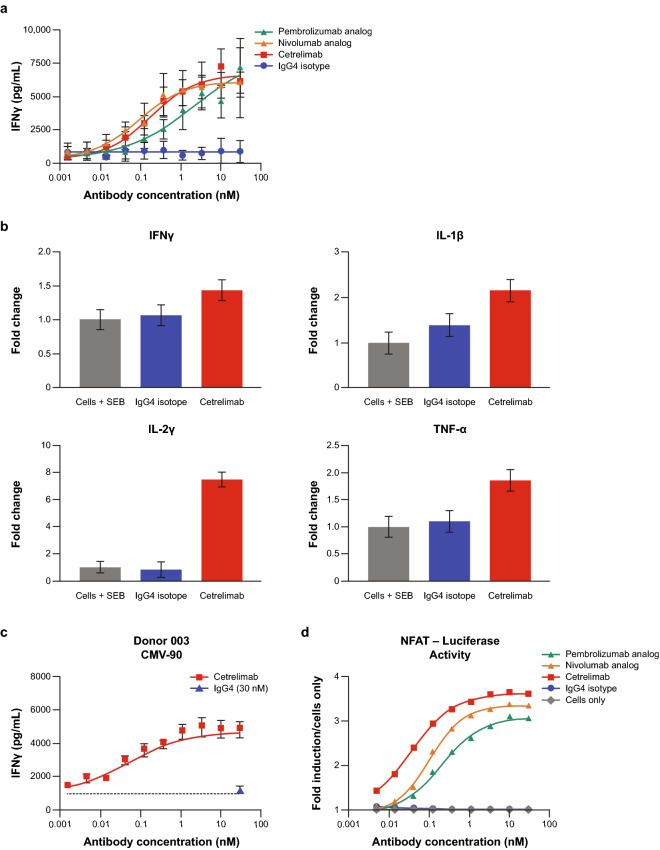
Cetrelimab promoted T cell activation and reversed PD-1-mediated suppression of T cell receptor signaling in vitro. **a** MLR assay. Purified human T cells (1.5 × 10^5^ cells) were activated by stimulation with allogeneic, major histocompatibility complex–mismatched, dendritic cells (5 × 10^3^ cells) for 5 days in the presence of cetrelimab, pembrolizumab analog, nivolumab analog, or isotype-control antibody. Data are from one representative experiment. Error bars represent SEM from one donor with one experiment in quintuplicate. **b** SEB assay. Isolated PBMCs (2 × 10^5^ cells) were stimulated with SEB and incubated with cetrelimab or isotype-control antibody for 3 to 6 days. Error bars represent the estimate of variability from the linear mixed-effect model for two donors, with each treatment in quadruplicate. *Significantly different from cells + SEB, *P* < 0.0001. **c** CMV recall. CMV-reactive T cells (1.5 × 10^5^ cells) were stimulated with CMV antigen for 6 days in the presence of cetrelimab or isotype-control antibody. Error bars represent SEM. **d** NFAT assay. Jurkat T cells (7.5 × 10^4^ cells) were incubated with PD-L1 positive Chinese hamster ovary-K1 cells (4 × 10^4^ cells) in the presence of cetrelimab, pembrolizumab analog, nivolumab analog, or isotype-control antibody for 24 h. Data are from one experiment. No error bars are shown as replicates were averaged and divided by the cells-only control. *CMV* cytomegalovirus, *IFN* interferon, *IL-* interleukin-, *MLR* mixed lymphocyte reaction, *NFAT* nuclear factor of activated T cells, *PBMC* peripheral blood mononuclear cell, *PD-1* programmed cell death protein-1, *SEB* staphylococcal enterotoxin B, *SEM* standard error of the mean, *TNF* tumor necrosis factor

In MLR assays, in which CD4^+^ T cells from healthy donors are activated by stimulation with allogeneic, MHC-mismatched dendritic cells for 5 days, cetrelimab increased IFN-γ (Fig. 2a). Concentration-dependent increases in IFN-γ were seen with cetrelimab, nivolumab analog, and pembrolizumab analog, although the extent of the response varied among donors. No enhancement of other cytokines was observed in response to cetrelimab treatment (data not shown). The robust EC_50_ values (*n* = 3 experiments × 3 donors) seen with cetrelimab were 0.08 ng/mL for IFN-γ, 0.07 ng/mL for IL-2, and 0.02 ng/mL for TNF-α, consistent with the pembrolizumab analog (0.05 ng/mL for IFN-γ, 0.03 ng/mL for IL-2, and 0.07 ng/mL for TNF-α) and the nivolumab analog (0.29 ng/mL for IFN-γ, 0.36 ng/mL for IL-2, and 0.03 ng/mL for TNF-α).

#### SEB stimulation

In human T cells obtained from healthy donors and exposed to super-antigen SEB, cetrelimab significantly enhanced IFN-γ, IL-1β, IL-2, and TNF-α secretion compared with SEB alone (*P* < 0.0001) (Fig. 2b). Increased release was specific to those four cytokines.

#### Antigen-specific recall

In CMV recall assays, cetrelimab treatment led to concentration-dependent induction of IFN-γ in CMV-stimulated T cells from a CMV-responsive human donor (Fig. 2c). The robust EC_50_s seen with cetrelimab were 0.03 ng/mL for IFN-γ, 0.08 ng/mL for IL-4, and 0.07 ng/mL for TNF-α. These values for nivolumab analog were 0.06 ng/mL for IFN-γ and 0.08 ng/mL for IL-4 (the TNF-α EC_50_ was uninterpretable due to model extrapolation); and for pembrolizumab analog were 0.03 ng/mL for IFN-γ, 0.01 ng/mL for IL-2, and 0.05 ng/mL for TNF-α. Cetrelimab treatment did not enhance levels for any of the other cytokines tested.

#### Reversal of PD-1-mediated TCR suppression

Cetrelimab treatment specifically reversed PD-1-mediated inhibition of TCR signaling as measured by a concentration-dependent increase in NFAT reporter activity at 24 h (Fig. 2d). This was also seen with nivolumab analog and pembrolizumab analog as evaluated. The robust EC_50_s for cetrelimab, nivolumab analog, and pembrolizumab analog were 0.50 ng/mL, 6.1 ng/mL, and 22.4 ng/mL, respectively.

### In vivo activity

#### Efficacy in hPD-1KI mice

**Fig. 3:**
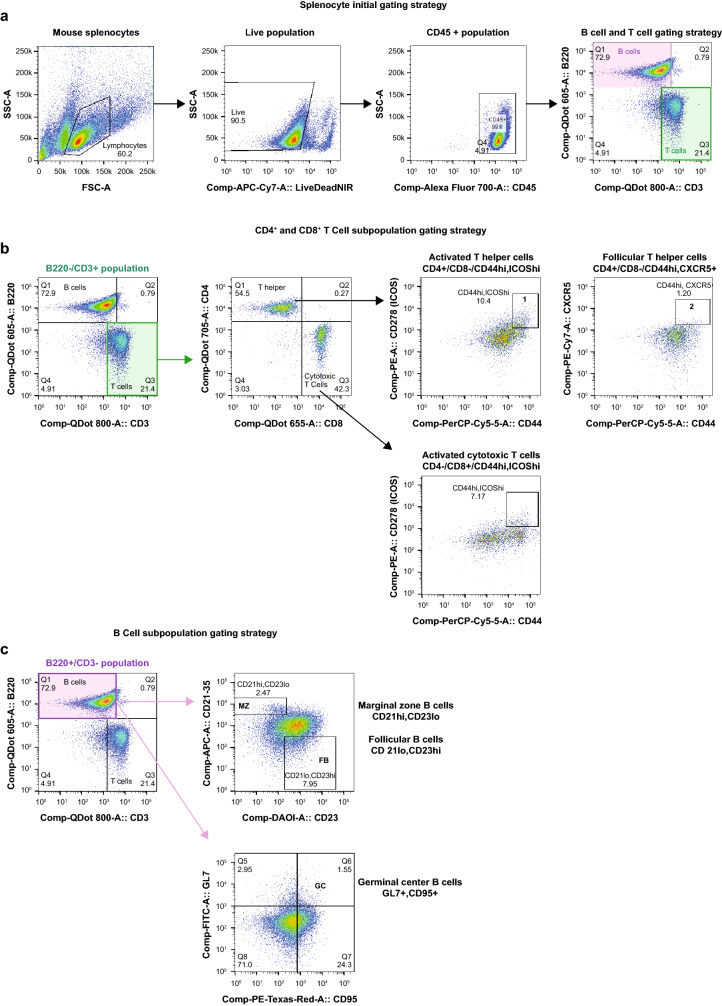
Phenotypic analysis of knock-in mouse splenocytes. Using previously optimized panels, 10 knock-in mouse splenocyte samples, previously isolated and stimulated (Janssen, Spring House, PA) were immunophenotyped (FlowMetric Life Science, Doylestown, PA). **a** Splenocyte initial gating strategy and B cell and T cell gating strategy. **b** T cell subpopulation gating strategy. **c** B cell subpopulation gating strategy. Plots show representative data for gating strategies

**Fig. 4:**
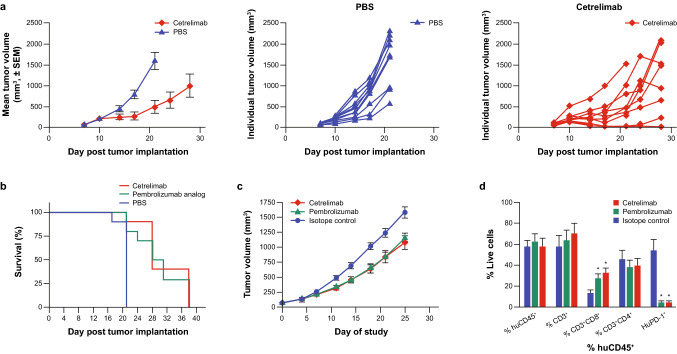
Cetrelimab showed antitumor efficacy in two in vivo models. **a** MC38 tumor growth inhibition in PD-1 knock-in mice. Error bars represent SEM, *n* = 10. **b** Survival of PD-1 knock-in mice bearing MC38 tumors following cetrelimab and pembrolizumab analog treatment, *n* = 10. **c** Patient-derived LG1306 lung tumor growth inhibition in huCD34^+^ NSG mice by cetrelimab and commercially available pembrolizumab. Error bars represent SEM, *n* = 10–11 from two donors. **d** Cetrelimab and commercially available pembrolizumab–enhanced peripheral blood T cells from in huCD34^+^-humanized NSG mice bearing established LG1306 PDX tumors. Error bars represent SEM, *n* = 10–11 from two donors. *P < 0.05 versus isotype-control–treated mice. *PBS* phosphate buffer saline, *PD-1* programmed cell death protein-1, *PDX* patient-derived xenograft, *SEM* standard error of the mean

Since cetrelimab is not cross-reactive to mouse PD-1, for in vivo testing we used hPD-1KI mice, in which the mouse PD-1 ECD was replaced with the human PD-1 ECD. These mice express a chimeric human/mouse PD-1 molecule (hPD-1), with human ECD and mouse transmembrane and intracellular PD-1 regions. The hPD-1KI mice develop normally and have no immune abnormalities (Fig. 3). Similar to endogenous mouse PD-1 in wild-type mice, hPD-1 is expressed on CD4^+^ and CD8^+^ T cells upon in vitro activation. For antitumor efficacy studies, hPD-1KI mice bearing established MC38 tumors were treated with cetrelimab or PBS control twice weekly. The mean MC38 tumor volume was significantly lower at Day 21 (*P* < 0.001) in animals treated with cetrelimab compared with the PBS control (Fig. 4a). Statistically significant survival benefits also occurred with cetrelimab and pembrolizumab analog treatments versus PBS control (*P* < 0.0001) (Fig. 4b).

#### PDX lung model

Tumor growth inhibition also was evaluated in the established LG1306 PDX model. Treatment with cetrelimab 10 mg/kg and commercially available pembrolizumab 10 mg/kg significantly reduced patient-derived tumor volume by 32% and 27%, respectively, compared with tumor volumes in mice treated with isotype control (*P* < 0.05) (Fig. 4c).

In addition, treatment with cetrelimab and commercially available pembrolizumab resulted in statistically significant increases in peripheral blood CD8^+^ T cells, with 33% and 28% of huCD45^+^ cells being CD3^+^CD8^+^, respectively, compared with 14% in the isotype-control–treated animals (*P* < 0.05; Fig. 4d). Moreover, in peripheral blood from isotype-control–treated animals, 54% of huCD45^+^ cells had PD-1 expression versus 5% in the cetrelimab– and commercially available pembrolizumab–treated groups. The apparent reduction of PD-1 by flow cytometry, due to the flow detection antibody competing with the therapeutic, demonstrates target engagement.

### Pharmacokinetics, immunogenicity, and toxicity of cetrelimab in cynos

#### Single-dose pharmacokinetics

A single IV administration of cetrelimab to cynos at 0.1, 1, and 10 mg/kg was well tolerated, with no effects on body weight or clinical observations. Maximum observed serum concentration (*C*_max_) of cetrelimab increased in an approximately dose-proportional manner, whereas area under the serum concentration–time curve from time 0 to infinity (AUC_inf_) increased in a greater than dose-proportional manner from 0.1 to 10 mg/kg (Table 1). The mean serum drug concentration–time profile in the 0.1 mg/kg dose group exhibited a steep decline over time while the mean profile from the 1 mg/kg dose group had a slower decline over time. The clearance decreased and terminal half-life (*T*_½_) increased as the dose increased from 0.1 to 1 mg/kg suggesting nonlinear PK of cetrelimab, possibly attributable to target-mediated drug deposition (TMDD). The clearance was similar between the dose levels of 1 and 10 mg/kg, suggesting saturation of TMDD within this range. In the 10 mg/kg group, large inter-animal variability and accelerated concentration decreases began at Day 15 for one male or Day 22 for one male and one female, likely due to ADAs (assay not conducted). As a result, the *T*_½_ from these animals could not be estimated accurately based on the quantifiable concentrations after Day 15 or Day 22. Compared with mean PK profiles in the 0.1 and 1 mg/kg groups, the profiles from all animals at 10 mg/kg displayed slower decline before Day 15. One female at 10 mg/kg exhibited a first-order drug concentration decrease up to Day 36 and then a fast concentration decrease after Day 36, which is consistent with TMDD rather than an ADA effect. For these reasons, in addition to *T*_½_ calculation using all available data, the *T*_½_ for the 10 mg/kg group was also determined based on the data before the accelerated concentration decrease; the mean *T*_½_ of 7.50 days may be closer to actual *T*_½_ of cetrelimab in cynos when TMDD is saturated.

**Table 1:** Serum cetrelimab toxicokinetic parameter estimates in cynomolgus monkeys administered a single intravenous dose

Dose (mg/kg)	Sex^a^	*C*_max_	AUC_last_	AUC_inf_	*CL*	*T*_½_	*T*_½_
		(µg/mL)	(µg∙day/mL)	(µg∙day/mL)	(mL/day/kg)	(day)	(day)
0.1	Male	1.33	1.57	2.50	40.66	2.30	–
	Female	1.56	1.91	2.07^b^	48.33^b^	1.31^b^	–
	Male and Female	1.45 (0.31)	1.74 (0.29)	2.36 (0.40)	43.22 (6.73)	1.97 (0.97)	–
1	Male	21.16	81.33	96.78	10.35	4.74	–
	Female	22.22	63.21	97.94	10.44	4.90	–
	Male and Female	21.69 (1.45)	72.27 (10.90)	97.36 (12.36)	10.40 (1.32)	4.82 (0.68)	–
10	Male	238.45	1132.68	1137.08	8.86	1.36	5.63^c^
	Female	251.52	1573.21	1574.31	6.37	2.89	9.36^d^
	Male and Female	244.99 (15.35)	1352.95 (275.15)	1355.69 (272.38)	7.61 (1.59)	2.12 (1.12)	7.50^c,d^ (2.18)

^a^*n* = 2 per sex except where noted. All values listed are mean, except male and female combined values are mean (± SEM)

^b^*n* = 1 due to poorly characterized terminal phase for the other animal

^c^*T*_½_ was calculated based on the time points up to Day 12 for one male and Day 15 for one male

^d^*T*_½_ was calculated based on the time points up to Day 15 for one female and Day 36 for one female

*AUC*_*inf*_ area under the concentration–time curve from time point 0 to infinity, *AUC*_*last*_ area under the concentration–time curve from time point 0 to the last quantifiable time point, *CL* clearance, *C*_*max*_ maximum serum concentration, *SEM* standard error of the mean, *T*_*½*_ terminal half-life

#### Repeated dose toxicokinetics

Similarly, cetrelimab was well tolerated in cynos over the 5-week treatment phase (cetrelimab IV 10, 30, or 100 mg/kg/week or placebo) and a 4-week post-injection period.

There were no effects on survival, observations (including feeding behavior), body weight, physical (including respiratory and neurologic assessments), electrocardiographic and ophthalmologic examinations, hematology, and serum chemistry parameters, or urinalysis parameters.

Mean serum concentration–time profiles indicated that most cynos had continuous exposure to cetrelimab at all doses throughout the dosing period that was sufficient to evaluate the toxicologic effects. In Week 5, mean *C*_max_ values and AUC exposures increased in an approximately dose-proportional manner from 10 to 100 mg/kg/week, were similar across sexes, and demonstrated accumulation (Table 2). Steady state was not fully reached by the last dose on Day 29. Of 30 cetrelimab-treated animals, 16 tested ADA positive. Drug exposure following repeated doses for 12 of 16 ADA-positive animals was lower than for ADA-negative animals in the same dose group. There were no apparent toxicologic impacts or sequelae due to ADAs.

**Table 2:** Mean serum cetrelimab toxicokinetic parameter estimates in cynomolgus monkeys administered five weekly intravenous doses

Dose (mg/kg)	Following dose on Day 1		Following dose on Day 29			
	*C*_max_^a^ (µg/mL)	AUC_Day1–8_^a^ (µg∙d/mL)	*C*_max_^a^ (µg/mL)	AUC_Day29–36_^a^ (µg∙d/mL)	R^b^	*T*_½_^c^ (day)
10	219.06	777.17	281.54	984.81	1.31	13.27 (*n* = 2)
30	622.41	2351.81	1037.64^d^	4446.90^d^	1.90	14.24 (*n* = 1)
100	1930.27	7270.58	3055.75	12,658.15	1.73	17.30 (*n* = 2)

^a^5 per sex per group, except where noted

^b^Mean of individual ratios

^c^Antidrug antibody–positive animals excluded for calculation of mean *T*_½_

^d^5 males + 4 females; not determined for 1 female with < 3 quantifiable data points

*AUC* area under the serum concentration–time curve, *C*_*max*_ maximum observed serum concentration, *R* accumulation ratio calculated from AUC_Day29–36_ after five doses and AUC_Day1–8_ after the first dose, *T*_*½*_ half-life

Cetrelimab (0, 10, 30, or 100 mg/kg/wk) with a 4-week post-injection period was well tolerated by cynos and the majority had continuous exposure to cetrelimab throughout the 5-week treatment period. Primary cetrelimab-related findings at ≥ 10 mg/kg/wk that were also generally consistent with pharmacology (immune stimulation) and/or target antigen (PD-1) tissue expression included reduced cellularity in the thymus (reduced lymphocytes) that was minimal in severity and did not correlate with gross or organ weight changes, slight prolongations in prothrombin times, minor decreases in absolute counts for CD3^+^ T, CD3^+^/CD4^+^ T-helper, and CD3^+^/CD8^+^ T-cytotoxic lymphocytes (only on Day 1 of the study), and increased IgM and IgG responses to a keyhole limpet hemocyanin antigen challenge (T cell–dependent antibody response) that were higher in cetrelimab-treated groups. All cetrelimab-related findings at the end of dosing were reversible following the 4-week recovery period. None of the cetrelimab-related findings were considered adverse; therefore, the no observed adverse effect level was 100 mg/kg/wk.

## Discussion

Cetrelimab is a fully human IgG4 kappa, hinge-stabilized mAb targeting PD-1 receptor ECD that was developed for use in combination regimens for the treatment of solid tumors. We have demonstrated that cetrelimab has higher affinity for human and cyno PD-1 ECD than PD-L1 binding to PD-1, based on K_D_ values. Anti-PD-1 antibodies approved for treatment of various cancers also showed higher binding affinities and specificities for PD-1 than the native interaction between PD-1 and its ligand [26–28]. Cetrelimab did not show significant binding to other members of the human PD-1 family, suggesting low potential for unwanted adverse events. There is no cross-reactivity with rat or mouse PD-1, allowing for assessment of the molecule in murine models of human tumors. In vitro assays showed that cetrelimab bound to transfected and endogenous PD-1 and to activated T cells, and blocked PD-1 ligand binding. Further, the cetrelimab binding profile translates to enhancement of T cell function in vitro. Cetrelimab treatment specifically increased T cell release of IFN-γ, IL-1β, IL-2, and TNF-2 and reversed PD-1-mediated TCR inhibition in a concentration-dependent manner, phenomena consistent with the established PD-1 inhibitors pembrolizumab and nivolumab.

In vivo, cetrelimab demonstrated antitumor activity in two tumor models [29, 30]. First, we used a hPD-1KI strain that expresses the human PD-1 ECD to test the efficacy of cetrelimab in a syngeneic MC38 model, shown in the literature to respond to PD-1 blockade. Cetrelimab significantly inhibited tumor growth and prolonged survival versus vehicle-treated controls in this model, consistent with results observed in pembrolizumab analog–treated mice. In addition, cetrelimab was also tested in a humanized PDX lung cancer model, in which it significantly inhibited tumor growth, consistent with the antitumor activity of commercially available pembrolizumab observed in this model. Cetrelimab treatment increased the number of peripheral CD8^+^ T cells and led to an apparent reduction of PD-1 levels on T cells, demonstrating PD activity and target engagement, respectively, consistent with effects observed with commercially available pembrolizumab treatment.

The PK of cetrelimab were evaluated in cynos after a single IV dose at 0.1, 1, or 10 mg/kg. For mAbs against cell surface target, target-mediated clearance can be a major route of elimination, especially at lower doses and concentrations [31]. Consistently, the mean (± SEM) clearance of cetrelimab decreased from 43.22 (± 6.73) to 10.40 (± 1.32) mL/kg/day when the dose increased from 0.1 to 1 mg/kg, suggesting presence of target-mediated clearance at doses below 1 mg/kg. However, the mean (± SEM) clearance of cetrelimab was similar between 1 and 10 mg/kg (10.40 ± 1.32 and 7.61 ± 1.59 mL/kg/day) doses, suggesting limited impact of target-mediated clearance at the clinically relevant dose ranges between 1 and 10 mg/kg. Toxicokinetics were evaluated in cynos after repeated IV doses of cetrelimab over 5 weeks and during a 4-week post-infusion follow-up. At doses that were adequate to maintain consistent exposure, cetrelimab was well tolerated. Although 16 of 30 cynos developed anti-cetrelimab antibodies, there were no toxicologic effects recorded, and it has been well established that immunogenicity in animal models is not predictive of immunogenicity in humans [32].

Toxicokinetics were evaluated in cynos after repeated IV doses of cetrelimab over 5 weeks and during a 4-week post-infusion follow-up. At doses that were adequate to maintain consistent exposure, cetrelimab was well tolerated. Although 16 of 30 cynos developed anti-cetrelimab antibodies, there were no toxicologic effects recorded.

Taken together, the data reported here support the ongoing first-in-human study of cetrelimab in patients with solid tumors, two phase 2 studies of cetrelimab in combination with TAR-200 in patients with NMIBC and MIBC, and a phase 3 study of cetrelimab in combination with TAR-200 versus concurrent chemoradiotherapy in patients with MIBC.

Data from the first-in-human, open label, phase 1/2 study of cetrelimab in patients with advanced cancers are reported in a companion paper in this volume of *Cancer Chemotherapy and Pharmacology*.

## Data Availability

The authors confirm that the data supporting the findings of this study are available within the article and its supplementary material.
